# New Insights Into Renal Involvement During Immune-Mediated Thrombotic Thrombocytopenic Purpura

**DOI:** 10.1016/j.ekir.2025.06.039

**Published:** 2025-06-23

**Authors:** Marie Robert, Valentin Maisons, Marion Rabant, Aude Servais, Charles Antunes, Nicolas Fage, Florent von Tokarski, Sophie Chauvet, Manon Dekeyser, Alain Wynckel, Anna Duval, Nadine Baroukh, Agnès Veyradier, Bérangère S. Joly, Paul Coppo, Jean-Michel Halimi, MATRIX Consortium Group and the MATHIS Group,, Isabelle Brocheriou-Spelle, Elodie Standley, Jerome Olagne, Nicolas Kozakowski, Ingrid Masson, Sophie Ferlicot, Charlotte Mussini, Hélène Perrochia, Vincent Vuiblet, Nolwenn Rabot, Jean-Paul Duong van Huyen, Viviane Gnemmi, Marie Robert, Valentin Maisons, Marion Rabant, Aude Servais, Charles Antunes, Nicolas Fage, Florent von Tokarski, Sophie Chauvet, Manon Dekeyser, Alain Wynckel, Anna Duval, Nadine Baroukh, Agnès Veyradier, Bérangère S. Joly, Paul Coppo, Jean-Michel Halimi

**Affiliations:** 1 Service d’Hématologie, Hôpital Saint-Antoine, Assistance Publique – Hôpitaux de Paris, Sorbonne Université, Paris, France; 2 Centre National de Référence des Microangiopathies Thrombotiques, Assistance Publique – Hôpitaux de Paris, Paris, France; 3 Service de Néphrologie, Hôpital Bretonneau, Centre de Référence pour les Microangiopathies Thrombotiques, CHU de Tours, Tours, France; 4 U1246, INSERM, SPHERE, Université de Tours, Université de Nantes, Tours, Nantes, France; 5 Pathology Department, AP-HP, Necker-Enfants Malades Hospital, Paris, France; 6 CNRS, Inserm, Institut Necker-Enfants Malades, Université Paris Cité, Paris, France; 7 Service de Néphrologie, Hôpital Necker, Assistance Publique – Hôpitaux Paris, Paris, France; 8 Service de Néphrologie, CHU de Brest, Brest, France; 9 Service de Néphrologie, Département de médecine intensive réanimation-médecine hyperbare, CHU d’Angers, Angers, France; 10 Service de Néphrologie, dialyse et transplantation rénale, Hôpital Foch, Suresnes, France; 11 Service de Néphrologie, Hôpital Européen Georges Pompidou, Assistance Publique – Hôpitaux Paris, Université Paris Cité, U1138 INSERM, FHU COMET, Paris, France; 12 Service de Néphrologie, CHU d’Orléans, Orléans, France; 13 Service de Néphrologie, CHU de Reims, Reims, France; 14 Service de Néphrologie, CHU de Strasbourg, Strasbourg, France; 15 UMR 1327, ISCHEMIA, Université de Tours, Tours, France; 16 Service d’Hématologie Biologique, Hôpital Lariboisière, AP-HP, Nord, Université Paris Cité, Paris, France; 17 INSERM Unité Mixte de Recherche S 1138, Centre de Recherche des Cordeliers, Université Paris Cité, Paris, France; 18 INI-CRCT, Tours, France

**Keywords:** biopsy, immune-mediated thrombotic thrombocytopenic purpura, kidney, thrombotic microangiopathies

## Introduction

Immune-mediated thrombotic thrombocytopenic purpura (iTTP) is a rare life-threatening thrombotic microangiopathy (TMA), driven by IgG autoantibodies against ADAMTS13, a protease responsible for cleaving von Willebrand factor. Unchecked ultralarge von Willebrand factor causes excessive platelet aggregation, microvascular occlusion, microangiopathic hemolytic anemia, and severe platelet consumption.^1^ Acute kidney injury (AKI) occurs in up to 60% of patients with iTTP^2^; however, its mechanism remains unclear. Candidate injuries include intratubular hemoglobin casts or acute tubular necrosis,^3^ glomerular endothelial damage caused by released heme, and local complement activation.^4^, ^5^, ^6^ Kidney biopsy is rarely performed because thrombocytopenia increases bleeding risk and AKI is often viewed as transient and moderate during iTTP. However, chronic kidney disease develops in a substantial proportion of patients.^2^ This highlights the need for more definitive histological data to elucidate the pathogenesis of iTTP-associated kidney involvement and to refine both management and prognostic assessments.

To address this gap, we examined kidney histology in iTTP using data from the French MATRIX consortium study, which included patients with TMA who underwent kidney biopsy (Supplementary Methods).^7^^,^^8^

## Results

### Baseline Characteristics

Among 1157 patients in the MATRIX Cohort, 10 (0.8%) had iTTP (Table 1, Supplementary Table S1). Six patients (60%) were women; median (interquartile range) age at kidney biopsy was 58 (52–64) years. Biopsy was performed a median of 18 (5–31) days after iTTP diagnosis. Three patients had an idiopathic iTTP and 7 had 1 or several associated condition: 6 patients (60%) had a history of autoimmune disease (2 with systemic lupus erythematosus; and 1 each with systemic sclerosis, pauci-immune vasculitis, Evans syndrome, and multiple sclerosis) and 2 patients had concomitant infections (1 with bacterial pneumonia and 1 infectious endocarditis). Malignant hypertension coexisted in 3 patients (30%). Laboratory results on admission to hospital are summarized in Supplementary Table S1. ADAMTS13 activity was < 10% in all patients and anti-ADAMTS13 IgG autoantibodies detected in 6 patients (60%).

**Table 1:** Individual data

Sex/Age (yr)	Biopsy year	Time between iTTP diagnosis and biopsy	Autoimmune disease	Infection	Idiopathic iTTP	Anti-ADAMTS13 Ab	Malignant hypertension	rTMA	Other lesions	TPE/PI	CS	IS	Outcome/serum creatinine
M/53	2012	15 d	Multiple sclerosis	–	–	+	+	AG	ATN	TPE	+	RTX	Transplantation
F/52	2009	16 d	Evans syndrome	–	–	–	–	AG	–	–	+	RTX	11 5 μmol/l
M/81	2010	5 d^a^	Pauci-immune vasculitis	–	–	–	–	–	Extracap. Prolif. GN Nephrosclerosis	TPE	+	RTX	Chronic dialysis/Death
F/35	2013	71 d	–	–	+	+	–	AG	Endocap. Prolif. GN	TPE	+	–	82 μmol/l
F/21	2021	28 d	SLE	–	–	+	–	G	Endocap Prolif. GN LN Class III+V	PI	+	MMF HCQ	46 μmol/l
F/57	2010	33 d	–	–	+	+	–	–	Interstitial nephritis	PI	+	–	99.4 μmol/l
F/59	2009	31 d	–	–	+	–	–	AG	ATN	TPE	+	–	76 μmol/l
M/59	2012	19 d	SLE	–	–	+	+	A	–	TPE	+	–	106 μmol/l
M/69	2017	0 d	–	Infectious endocarditis^b^	–	–	–	–	Extracap. Prolif. GN	TPE	–	–	Death–175 μmol/l
F/65	2016	5 d	SSc	Lung infection^c^	–	+	+	A	Malignant nephrosclerosis	TPE	+	RTX	Chronic dialysis/death

A, arteriolar lesions; Ab, antibodies; AG, arteriolar and glomerular lesions; ATN, acute tubular necrosis; CS, corticosteroids; Endocap. Prolif. GN, endocapillary proliferative glomerulonephritis; Extracap. Prolif. GN, extracapillary proliferative glomerulonephritis; F, female; G, glomerular lesions; HCQ, hydroxychloroquine; IS, immunosuppressive therapies; iTTP, immune-mediated thrombotic thrombocytopenic purpura; LN, lupus nephritis; M, male; MMF, mycophenolate mofetil; PI, plasma infusions; rTMA, renal TMA; RTX, rituximab; SLE, systemic lupus erythematosus; SSc, systemic sclerosis; TPE, therapeutic plasma exchanges.

Idiopathic iTTP refers to patients with iTTP without associated condition including history of auto-immune disease or concomitant infection.

a Means that the biopsy was performed before iTTP diagnosis. All patients had hematologic features of thrombotic microangiopathy.

b Infectious endocarditis was due to *Streptococcus sanguinis*.

c Lung infection was secondary to *Moraxella catarrhalis* and *Escherichia coli* infection.

### Pathological Findings

**Figure 1:**
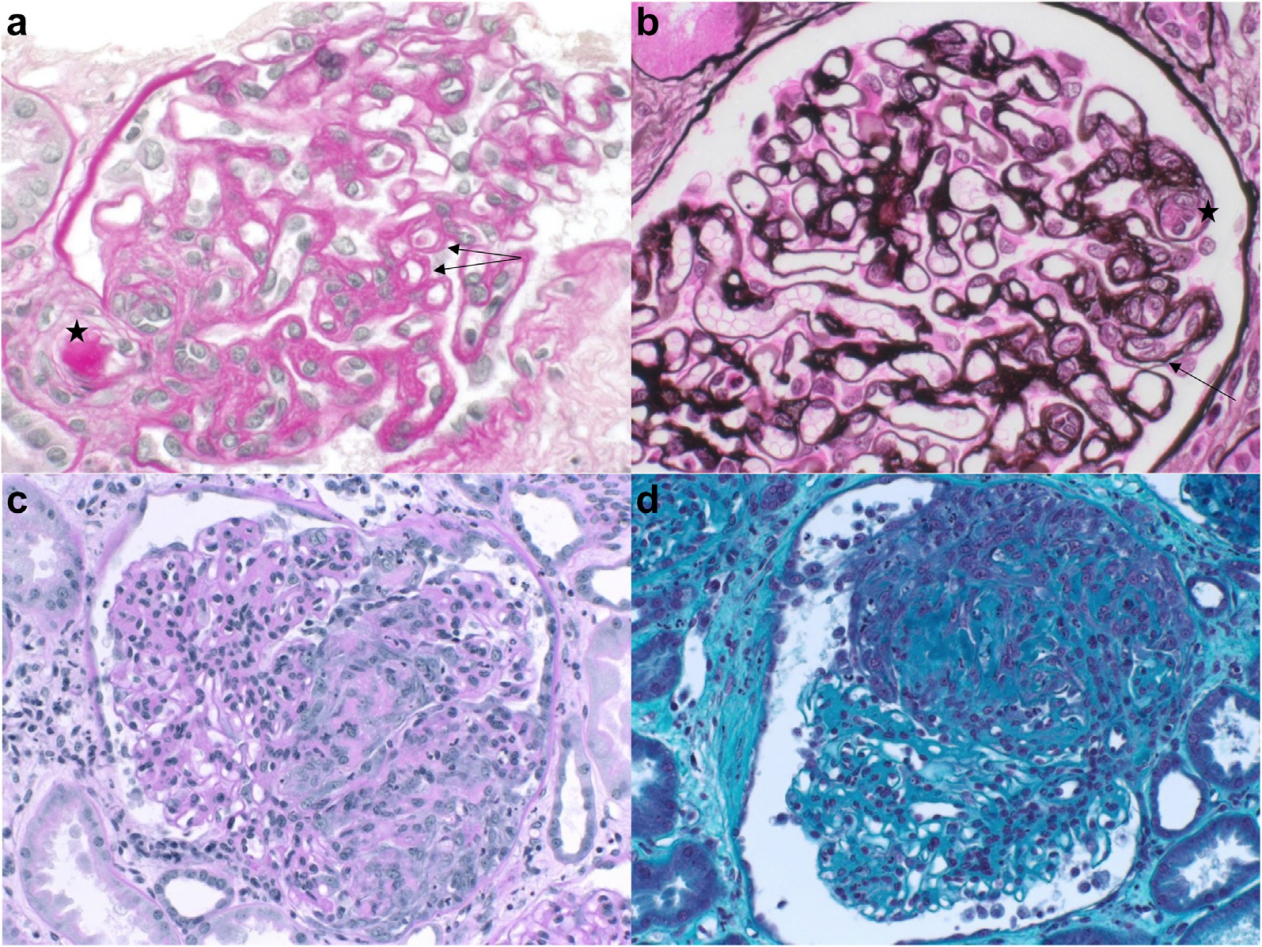
Representative images of 2 patients with immune-mediated thrombotic thrombocytopenic purpura. (1) Patient with renal TMA in the context of systemic sclerosis: (a) arteriolar thrombosis in the preglomerular arteriole (star), and double contours (arrow) (PAS staining, x40); (b) endothelial swelling (star) and double contours (arrow) (Jones staining, x40). (2) Patient with nonrenal TMA in the context of endocarditis: (c) (PAS staining x20) and (d) (Masson Trichrome staining x20) showing glomeruli with mesangial hypertrophy and hypercellularity, endocapillary hypercellularity with neutrophils, and cellular crescent with fibrinoid necrosis. PAS, periodic acid-schiff; TMA, thrombotic microangiopathy.

Kidney biopsies were indicated for persistent renal insufficiency despite the resolution of hematologic features of TMA (*n* = 6) or to exclude differential diagnoses in patients with systemic lupus erythematosus (*n* = 2), systemic sclerosis (*n* = 1) (Figure 1a and b), or endocarditis (*n* = 1) (Figure 1c and d). In patients with iTTP with an associated condition, the median time between iTTP diagnosis and renal biopsy was significantly shorter than in patients with idiopathic iTTP (15 [0–19] days vs. 33 [31–71] days, *P* < 0.05) (Supplementary Table S2). Renal TMA (rTMA) was found in 7 patients (70%): 4 showed combined arteriolar and glomerular lesions, 2 with isolated arteriolar lesions, and 1 with isolated glomerular TMA lesions (Supplementary Tables S1 and S3). Additional histopathological findings were as follows: mild mesangial hypercellularity with negative immunofluorescence findings (*n* = 1), lupus nephritis class 3 and 5 with mesangial hypercellularity and endocapillary proliferation (*n* = 1), acute tubular necrosis lesions (*n* = 2), and arteriosclerosis lesions with intima hyperplasia (*n* = 1). Among the 3 patients without rTMA, kidney biopsy findings included mild acute tubular necrosis (*n* = 1), pauci-immune vasculitis (*n* = 1) and per-infectious glomerulonephritis in the context of endocarditis (*n* = 1) (Supplementary Table S3).

### Management and Outcomes

Five patients required intensive care admission; 4 needed acute dialysis, and 3 patients eventually required chronic dialysis. Therapeutic plasma exchanges were performed in 7 patients (70%), plasma infusions in 2 (20%). All but 1 (90%) received corticosteroids; rituximab was administered to 4 patients and mycophenolate mofetil was continued in 1 patient. Neither complement inhibitors nor caplacizumab were administered. During hospitalization, 5 (patients 50%) experienced major adverse cardiovascular events, but none died acutely. After a median follow-up of 37 (18–88) months, 3 patients died (cardiac arrest, myocardial infarction, and hypovolemic and cardiogenic shock in the context of pseudomembranous rectitis), 2 patients remained on chronic dialysis, and 1 underwent kidney transplantation (Table 1).

## Discussion

This study provides novel insights into AKI associated with iTTP. Our results suggest the frequent association between iTTP and another autoimmune or infectious diseases when AKI is present. In most cases, biopsy revealed rTMA, even in patients with idiopathic iTTP, predominantly showing both arteriolar and glomerular lesions. Notably, among idiopathic patients with iTTP, none required chronic dialysis or kidney transplantation by the end of follow-up. These observations suggest that AKI may be a consequence of rTMA, which affects both the arterioles and glomeruli.

In the settings of TMA, kidney biopsies are rarely performed when a diagnosis of iTTP is established as not recommended. Herein, biopsies were performed either to rule out differential diagnoses or because of persistent AKI after the resolution of hematological TMA. For instance, the 3 idiopathic iTTP patients underwent kidney biopsies at least 1 month after the diagnosis because of prolonged AKI. In addition, nephrotoxicity of free iron, leading to renal sideropathy, was considered as the probable cause of AKI during iTTP.^9^ However, in contrast to common belief, acute tubular necrosis was observed in fewer than one-third of our patients, whereas rTMA was the predominant finding, regardless of an associated condition. The distinction between patients with idiopathic iTTP and the ones with autoimmune disease or infection was made to decipher the specific histopathological lesions of iTTP and its renal prognosis. Indeed, both arterioles and glomeruli were affected when rTMA occurred in patients with idiopathic iTTP, whereas in patients with associated conditions, either glomerular or arteriolar lesions were observed. In addition, complement activation has been proposed as a potential contributor to kidney vascular damage.^7^ Low C3 level was documented in 3 of 7 tested patients, aligning with previous reports showing that 30% of patients with iTTP with AKI had decreased C3 level, compared with only 8% without AKI.^2^ Genetic testing was not assessed in this small group. Any mechanistic interpretation remains speculative at this stage; however, future studies are needed to clarify whether and how complement activation, particularly at the level of the glomeruli, may drive kidney injury in iTTP. In addition, even if our cohort predates the triplet regimen era (therapeutic plasma exchanges, immunosuppression, and caplacizumab), a striking observation is that no patient with idiopathic iTTP required chronic dialysis or kidney transplantation at the last follow-up. In patients with additional triggers, kidney replacement therapy was occasionally required (see Supplementary References S1-S3).

Limitations of this study include the small sample size inherent to the rarity of kidney biopsies in iTTP, the selection bias toward patients with persistent AKI or diagnostic uncertainty, the temporal heterogeneity (pre-caplacizumab era) of our cohort, the high proportion of iTTP patients with associated condition, and the lack of central and blinded pathology review. Consequently, drawing firm conclusions about renal prognosis under the modern triplet regimen is challenging and prevents the generalizability of our findings to the full spectrum of patients with iTTP. Nevertheless, we believe that reporting these 10 cases provides valuable insights into the mechanisms of AKI in iTTP.

In conclusion, our findings suggest that renal involvement in iTTP is primarily secondary to rTMA and remains a significant challenge, requiring a multidisciplinary approach for optimal management.

## Appendix

### List of the members of the MATRIX Consortium Group and MATHIS Group

**MATRIX Consortium Group:** Marie Robert, Valentin Maisons, Marion Rabant, Aude Servais, Charles Antunes, Nicolas Fage, Florent von Tokarski, Sophie Chauvet, Manon Dekeyser, Alain Wynckel, Anna Duval, Nadine Baroukh, Agnès Veyradier, Bérangère S. Joly, Paul Coppo, and Jean-Michel Halimi.

**MATHIS Group:** Isabelle Brocheriou-Spelle, Elodie Standley, Jerome Olagne, Nicolas Kozakowski, Ingrid Masson, Sophie Ferlicot, Charlotte Mussini, Hélène Perrochia, Vincent Vuiblet, Nolwenn Rabot, Jean-Paul Duong van Huyen, and Viviane Gnemmi.

## Disclosure

MR is a fellow of the Ecole de l’Inserm Liliane Bettencourt. AV is a member of the French Advisory boards for caplacizumab (Sanofi) and recombinant ADAMTS13 (Takeda). BSJ is a member of the French advisory boards for Alexion and received speaker fees from Sanofi, Takeda. PC is a member of the Clinical Advisory Board for Alexion, Sanofi and Takeda. JMH reports receiving honoraria for lectures and travel grant from Alexion and Astra Zeneca. All the other authors declared no competing interests.

## Supplementary Material

Supplementary File (PDF) **Supplementary Methods. Supplementary References. Table S1.** Characteristics of patients according to the presence of renal thrombotic microangiopathy (rTMA). **Table S2.** Characteristics of patients according to concomitant disease to immune-mediated thrombotic thrombocytopenic purpura (iTTP). **Table S3.** Histologic evaluation of each biopsy. STROBE Checklist.

## Footnotes

Supplementary File (PDF)

**Supplementary Methods.**

**Supplementary References.**
**Table S1.** Characteristics of patients according to the presence of renal thrombotic microangiopathy (rTMA). **Table S2.** Characteristics of patients according to concomitant disease to immune-mediated thrombotic thrombocytopenic purpura (iTTP). **Table S3.** Histologic evaluation of each biopsy. STROBE Checklist.
