# Supplementary material for: New Insights Into Renal Involvement During Immune-Mediated Thrombotic Thrombocytopenic Purpura
Source: Kidney Int Rep. 2025 Jun 23;10(9):3271–5. doi: 10.1016/j.ekir.2025.06.039 (PMC12446935; doi:10.1016/j.ekir.2025.06.039)
Supplement: Supplementary File (PDF) — Supplementary Methods. Supplementary References. Table S1. Characteristics of patients according to the presence of renal thrombotic microangiopathy (rTMA). Table S2. Characteristics of patients according to concomitant disease to immune-mediated thrombotic thrombocytopenic purpura (iTTP). Table S3. Histologic evaluation of each biopsy. STROBE Checklist. [file mmc1.pdf]

SUPPLEMENTARY METHODS

iTTP patients were identified within The French Cohort MATRIX.[7] iTTP diagnosis required findings of TMA with ADAMTS13 activity < 10%.[8] Idiopathic iTTP patients referred to patients without a documented history of autoimmune disease or evidence of active or significant infection at the time of TMA diagnosis. Further details about methods are available in the **Supplementary Methods**.

Supplementary Methods

Study design and Patient selection

The MATRIX Consortium study is a large, multicenter French cohort conducted across 32 nephrology departments. It includes patients admitted to hospitals between January 1, 2009 and 2024, provided they met the following criteria: age ≥ 18 years, biopsy of a native kidney and presence of thrombotic microangiopathy (TMA). Among the 1,157 patients currently included, 10 (0.8%) were identified as having immune-mediated thrombotic thrombocytopenic purpura (iTTP). The diagnosis of iTTP was based on the presence of TMA findings with ADAMTS13 activity <10%.[S1]

Data collected

Patients underwent kidney biopsy because of acute kidney injury. Native kidney biopsies were analyzed by local renal pathologists and histologic reports were independently reviewed in a double-bind manner by 2 nephrologists (VM and JMH) and one pathologist (MRa). The evaluation included glomerular, vascular and tubular disease. Renal-limited TMA was defined on consensual criteria established by local renal pathologists.[S2] The presence of TMA with hematological features (RH-TMA) was determined by physicians at each participating center using platelet count, hemoglobin level, lactate dehydrogenase level, schistocytosis and haptoglobin level on admission to hospital.[S3] The underlying cause of TMA was classified by clinicians according to KDIGO criteria.[S2] Patient medical history, biological data (on admission to hospital), presence of malignant hypertension, TMA- and iTTP-related treatments, and in-hospital major events (major adverse cardiovascular events [MACE], intensive care unit [ICU] admission) were recorded. Additionally, the need for acute or chronic dialysis, renal transplantation, and mortality outcomes were recorded.

Ethics

Ethical approval was obtained from the Centre Val de Loire Region Ethics Committee (no. 2022-59). The study was approved by the French National Commission for Data Protection and Liberties (Commission Nationale de l'Informatique et des Libertés [CNIL]) protecting human rights in France (registration no. F20221110095846). Patients were informed that their medical records might be used for research in accordance with the privacy rule. The study protocol is registered on clinicaltrials.gov (NCT05991245).

Statistical analyses

Qualitative data are represented as number (percentages) and quantitative data as median (interquartile range, IQR). Analyses were carried out using R software (version 4.3.1).

Supplementary Table 1. Characteristics of patients according to the presence of renal thrombotic microangiopathy (rTMA)

**Characteristic**	**N**	**Overall**, N = 10	**rTMA**, N = 7	**No rTMA**, N = 3
**General characteristics**				
Age at kidney biopsy, median (IQR), years	10	58 (52–64)	53 (44–59)	69 (63–75)
Time between iTTP diagnosis and kidney biopsy, median (IQR), days	10	18 (5–31)	19 (15–31)	0 (-5*–33)
Male sex, n (%)	10	4 (40)	2 (29)	2 (66)
History of autoimmune disease, n (%)	10	6 (60)	5 (71)	1 (33)
Idiopathic iTTP, n (%)	10	3 (30)	2 (29)	1 (33)
Concomitant infection, n (%)	10	2 (20)	1 (14)	1 (33)
Malignant hypertension, n (%)	10	3 (30)	3 (43)	0
**Biological characteristics**				
Hemoglobin, median (IQR), g/L	10	90 (65–91)	73 (61–92)	90 (90–91)
Platelet count, G/L	10	52 (48–86)	53 (35–91)	51 (50–67)
Schistocytes positive, n (%)	10	8 (80)	7 (100)	1 (33)
Low haptoglobin, n (%)	10	9 (90)	6 (86)	3 (100)
Lactate deshydrogenase, median (IQR), xULN	8	2.3 (1.7–2.7)	2.6 (2.0–2.6)	1.7 (1.5–2.7)
Serum creatinine, median (IQR), µmol/L	10	163 (122–373)	184 (111–390)	141 (135–275)
Proteinuria, median (IQR), g/g	10	1.1 (0.8–3.3)	1.2 (0.7–2.9)	1.0 (1.0–17)
Prothrombin time, median (IQR), %	7	84 (78–89)	88 (84–98)	73 (73–80)
Fibrinogen, median (IQR), g/L	7	2.5 (2.2–4.1)	3.9 (2.4–5.4)	2.2 (1.9–2.6)
Low C3 level, n (%)	7	3 (43)	1 (25)	2 (67)
Low C4 level, n (%)	7	2 (29)	1 (25)	1 (33)
**Histopathological findings**				
Renal TMA lesions, n (%)	10	7 (70)	7 (100)	0
Arteriolar only		2 (20)	2 (29)	–

**Characteristic**	**N**	**Overall**, N = 10	**rTMA**, N = 7	**No rTMA**, N = 3
Glomerular only		1 (10)	1 (14)	–
Arteriolar and glomerular		4 (40)	4 (57)	–
Other lesions, n (%)	10			
Glomerular		3 (30)	1 (14)	2 (66)
Tubulointerstitial inflammation		1 (10)	0	1 (33)
Acute tubular necrosis		3 (30)	2 (29)	1 (33)
Vascular		2 (20)	1 (14)	1 (33)
**Events during hospitalization**, n (%)				
MACE	10	5 (50)	2 (29)	3 (100)
Acute dialysis	10	4 (40)	3 (43)	1 (33)
Chronic dialysis	10	3 (30)	2 (29)	1 (33)
Hospitalization in ICU	10	5 (50)	4 (57)	1 (33)
Death	10	0	0	0
**Treatments, n (%)**				
Plasma exchange	10	7 (70)	5 (71)	2 (67)
Plasma infusions	10	2 (20)	1 (14)	1 (33)
Steroids	10	9 (90)	7 (100)	2 (67)
Immunosuppressive therapy	10	5 (50)	4 (57)	1 (33)
Anti-hypertensive	10	4 (40)	4 (57)	0
Anticoagulation therapy	10	1 (10)	1 (14)	0
**Events during follow-up**				
Duration of follow-up, median (IQR), months	10	37 (18–88)	63 (26–113)	32 (18–34)
Chronic dialysis, n (%)	10	2 (20)	1 (14)	1 (33)
Kidney transplantation, n (%)	10	1 (10)	1 (14)	0
Death, n (%)	10	3 (30)	1 (14)	2 (67)

rTMA: renal thrombotic microangiopathy; iTTP: immune-mediated thrombotic thrombocytopenic purpura; MACE: major adverse cardiovascular events; ICU: intensive care unit. Idiopathic iTTP refers to patients with iTTP without associated condition including history of auto-immune disease or concomitant infection. * is a negative value as the biopsy was performed before iTTP diagnosis in one patient.

Supplementary Table 2. Characteristics of patients according to concomitant disease to immune-mediated thrombotic thrombocytopenic purpura (iTTP).

Characteristic	N	**iTTP + associated disease**, N = 7	**Idiopathic iTTP**, N = 3
**General characteristics**			
Age at kidney biopsy, median (IQR), years	10	59 (53–67)	57 (46–58)
Time between iTTP diagnosis and kidney biopsy, median (IQR), days[†]	10	15 (0–19)*	33 (31–71)
Male sex, n (%)	10	4 (57)	0
History of autoimmune disease, n (%)	10	6 (86)	0
Concomitant infection, n (%)	10	2 (29)	0
Malignant hypertension, n (%)	10	3 (43)	0
**Biological characteristics**			
Hemoglobin, median (IQR), g/L	10	73 (61–91)	90 (90–98)
Platelets, G/L	10	82 (51–91)	48 (35–50)
Schistocytes positive, n (%)	10	5 (71)	3 (100)
Low haptoglobin, n (%)	10	6 (86)	3 (100)
Lactate deshydrogenase, median (IQR), xULN	8	2.0 (1.6–2.6)	3.6 (3.6–3.6)
Serum creatinine, median (IQR), µmol/L	10	128 (111–462)	184 (163–225)
Proteinuria, median (IQR), g/g	10	2.3 (1.1–4.5)	0.7 (0.4–0.8)
Prothrombin time, median (IQR), %	7	83 (75–89)	86 (86–86)
Fibrinogen, median (IQR), g/L	7	2.4 (2.2–4.6)	2.9 (2.9–2.9)
Low C3 level, n (%)	7	3 (75)	0
Low C4 level, n (%)	7	2 (50)	0
**Histopathological findings**			
Renal TMA lesions, n (%)	10	5 (71)	2 (67)
Arteriolar only		2 (29)	0

**Characteristic**	**N**	**iTTP + associated disease**, N = 7	**Idiopathic iTTP**, N = 3
Glomerular only		1 (14)	0
Arteriolar and glomerular		2 (29)	2 (67)
Other lesions, n (%)	10		
Glomerular		2 (29)	1 (33)
Tubulointerstitial inflammation		0	1 (33)
Acute tubular necrosis		2 (29)	1 (33)
Vascular		2 (29)	0
**Events during hospitalization**, n (%)			
MACE	10	3 (43)	2 (67)
Acute dialysis	10	3 (43)	1 (33)
Chronic dialysis	10	3 (43)	0
Hospitalization in ICU	10	3 (43)	2 (67)
Death	10	0	0
**Treatments, n (%)**			
Plasma exchange	10	5 (71)	2 (67)
Plasma infusions	10	1 (14)	1 (33)
Steroids	10	6 (86)	3 (100)
Immunosuppressive therapy	10	5 (71)	0
Anti-hypertensive	10	4 (57)	0
Anticoagulation therapy	10	0	1 (33)
**Events during follow-up**			
Duration of follow-up, median (IQR), months	10	36 (9–83)	63 (47–79)
Chronic dialysis, n (%)	10	2 (29)	0
Kidney transplantation, n (%)	10	1 (14)	0
Death, n (%)	10	3 (43)	0

rTMA: renal thrombotic microangiopathy; iTTP: immune-mediated thrombotic thrombocytopenic purpura; MACE: major adverse cardiovascular events; ICU: intensive care unit. Idiopathic iTTP refers to patients with iTTP without associated condition including history of auto-immune disease

or concomitant infection. * in this group, one patient had a negative value as the biopsy was performed before iTTP diagnosis. †This comparison showed a significant difference, $p < 0.05$.

Supplementary Table 3. Histologic evaluation of each biopsy.

Sex/Age	Biopsy indication	Glomeruli number	Sclerotic glomeruli	Glomerular lesions	Tubulointerstitial lesions	Vascular lesions	IF findings	Final diagnosis
M/53	AKI	10	0	Ischemic glomeruli<br>TMA (double contours)	IF/TA 1<br>ATN	Arteriolar TMA (thrombosis, myxoid aspects)	Negative	Acute glomerular and arteriolar TMA
F/52	Creatinine 125 µmol/L with proteinuria 1.3g/d and hematuria	20	6	Ischemic glomeruli	IF/TA 2	Chronic arteriolar TMA (occlusion)	Negative	Chronic arteriolar TMA
M/81	AKI with anuria, biological TMA, urinary tract infection	20	3	Extracapillary proliferation<br>FSGS lesions	IF/TA 2	Intimal hyperplasia	Negative	Pauci-immune vasculitis
F/35	CKD with proteinuria 0.5g/L, no hematuria.	33	1	Ischemic glomeruli<br>Double contours<br>Inflammatory infiltrates	Cortical atrophy<br>IF/TA 2	Arteriolar thrombosis<br>Intimal hyperplasia	Negative	Acute glomerular and arteriolar TMA
F/21	Nephrotic syndrome, no hematuria in context of SLE	50	0	Endocapillary proliferation<br>Mesangial hypercellularity<br>TMA (mesangiolysis)	No	No	Full house deposits	Lupus nephritis class III and V<br>+<br>Acute glomerular TMA
F/57	AKI	7	1	No	IF/TA 1<br>ATN	No	Negative	ATN
F/59	AKI with oliguria, seizure<br>Proteinuria 0.55g/d	5	0	Ischemic glomeruli<br>TMA (mesangiolysis, double contours)	ATN	Arteriolar TMA (thrombosis, myxoid aspects)	Negative	Acute glomerular and arteriolar TMA
M/59	AKI in context of SLE<br>Proteinuria 0.5g/d	20	2	Ischemic glomeruli<br>Double contours<br>Mild mesangial hypercellularity<br>Segmental lesion	IF/TA 1	Chronic arteriolar TMA (occlusion, onion skin)	Negative	Acute and chronic glomerular and arteriolar TMA
M/69	AKI, proteinuria 0.5g/g, hematuria, purpura, ANCA positive, decreased complement	14	0	Extra- and endocapillary proliferation<br>Mesangial hypercellularity	IF/TA 1	Chronic vascular lesions (severe intimal hyperplasia, arteriolar hyalinosis)	C3 predominant	Per infectious glomerulonephritis secondary to endocarditis
F/65	AKI, biological TMA, antiScl70 autoantibodies positive	15	0	Ischemic glomeruli	Cortical necrosis	Arteriolar TMA (thrombosis, onion skin, myxoid)	Negative	Acute arteriolar TMA

AKI: acute kidney Injury; CKD: chronic kidney disease; TMA: thrombotic microangiopathy; IF/TA: interstitial fibrosis and tubular atrophy; ATN: acute tubular necrosis; SLE: systemic lupus erythematosus.

Supplementary references

S1. Coppo, P. et al. Predictive features of severe acquired ADAMTS13 deficiency in idiopathic thrombotic microangiopathies: the French TMA reference center experience. *PLoS One* **5**, e10208 (2010).

S2. Goodship, T.H. et al. Atypical hemolytic uremic syndrome and C3 glomerulopathy: conclusions from a "Kidney Disease: Improving Global Outcomes" (KDIGO) Controversies Conference. *Kidney Int* **91**, 539-551 (2017).

S3. Brocklebank, V., Wood, K.M. & Kavanagh, D. Thrombotic Microangiopathy and the Kidney. *Clin J Am Soc Nephrol* **13**, 300-317 (2018).

STROBE Statement—checklist of items that should be included in reports of observational studies

	Item No.	Recommendation	Page No.	Relevant text from manuscript
**Title and abstract**	1	(*a*) Indicate the study's design with a commonly used term in the title or the abstract	1	
		(*b*) Provide in the abstract an informative and balanced summary of what was done and what was found	N/A	
		**Introduction**		
Background/rationale	2	Explain the scientific background and rationale for the investigation being reported	3	
Objectives	3	State specific objectives, including any prespecified hypotheses	3	
		**Methods**		
Study design	4	Present key elements of study design early in the paper	4	
Setting	5	Describe the setting, locations, and relevant dates, including periods of recruitment, exposure, follow-up, and data collection	Supp p1	
Participants	6	(*a*) *Cohort study*—Give the eligibility criteria, and the sources and methods of selection of participants. Describe methods of follow-up<br>*Case-control study*—Give the eligibility criteria, and the sources and methods of case ascertainment and control selection. Give the rationale for the choice of cases and controls<br>*Cross-sectional study*—Give the eligibility criteria, and the sources and methods of selection of participants	4 and Supp p1	
		(*b*) *Cohort study*—For matched studies, give matching criteria and number of exposed and unexposed<br>*Case-control study*—For matched studies, give matching criteria and the number of controls per case		
Variables	7	Clearly define all outcomes, exposures, predictors, potential confounders, and effect modifiers. Give diagnostic criteria, if applicable	4	
Data sources/ measurement	8*	For each variable of interest, give sources of data and details of methods of assessment (measurement). Describe comparability of assessment methods if there is more than one group	Supp p1	
Bias	9	Describe any efforts to address potential sources of bias	7	
Study size	10	Explain how the study size was arrived at	4 and Supp p1	

Continued on next page

Quantitative variables	11	Explain how quantitative variables were handled in the analyses. If applicable, describe which groupings were chosen and why	Supp p2
Statistical methods	12	(*a*) Describe all statistical methods, including those used to control for confounding	Supp p2
		(*b*) Describe any methods used to examine subgroups and interactions	Supp p2
		(*c*) Explain how missing data were addressed	N/A
		(*d*) *Cohort study*—If applicable, explain how loss to follow-up was addressed<br>*Case-control study*—If applicable, explain how matching of cases and controls was addressed<br>*Cross-sectional study*—If applicable, describe analytical methods taking account of sampling strategy	N/A
		(*e*) Describe any sensitivity analyses	N/A
		**Results**	
Participants	13*	(a) Report numbers of individuals at each stage of study—eg numbers potentially eligible, examined for eligibility, confirmed eligible, included in the study, completing follow-up, and analysed	Supp p1
		(b) Give reasons for non-participation at each stage	N/A
		(c) Consider use of a flow diagram	N/A
Descriptive data	14*	(a) Give characteristics of study participants (eg demographic, clinical, social) and information on exposures and potential confounders	4
		(b) Indicate number of participants with missing data for each variable of interest	Supp p3-4
		(c) *Cohort study*—Summarise follow-up time (eg, average and total amount)	Supp p4-6
Outcome data	15*	*Cohort study*—Report numbers of outcome events or summary measures over time	N/A
		Case-control study—Report numbers in each exposure category, or summary measures of exposure	
		Cross-sectional study—Report numbers of outcome events or summary measures	
Main results	16	(*a*) Give unadjusted estimates and, if applicable, confounder-adjusted estimates and their precision (eg, 95% confidence interval). Make clear which confounders were adjusted for and why they were included	N/A
		(*b*) Report category boundaries when continuous variables were categorized	N/A
		(*c*) If relevant, consider translating estimates of relative risk into absolute risk for a meaningful time period	N/A

Continued on next page

Other analyses	17	Report other analyses done—eg analyses of subgroups and interactions, and sensitivity analyses	N/A
		**Discussion**	
Key results	18	Summarise key results with reference to study objectives	6-7
Limitations	19	Discuss limitations of the study, taking into account sources of potential bias or imprecision. Discuss both direction and magnitude of any potential bias	7
Interpretation	20	Give a cautious overall interpretation of results considering objectives, limitations, multiplicity of analyses, results from similar studies, and other relevant evidence	7
Generalisability	21	Discuss the generalisability (external validity) of the study results	7
**Other information**			
Funding	22	Give the source of funding and the role of the funders for the present study and, if applicable, for the original study on which the present article is based	8

*Give information separately for cases and controls in case-control studies and, if applicable, for exposed and unexposed groups in cohort and cross-sectional studies.

Note: An Explanation and Elaboration article discusses each checklist item and gives methodological background and published examples of transparent reporting. The STROBE checklist is best used in conjunction with this article (freely available on the Web sites of PLoS Medicine at http://www.plosmedicine.org/, Annals of Internal Medicine at http://www.annals.org/, and Epidemiology at http://www.epidem.com/). Information on the STROBE Initiative is available at www.strobe-statement.org.
